# Grip strength ratio: a grip strength measurement that correlates well with DASH score in different hand/wrist conditions

**DOI:** 10.1186/1471-2474-15-336

**Published:** 2014-10-06

**Authors:** Annechien Beumer, Tommy R Lindau

**Affiliations:** Upper Limb Unit of the Department of Orthopaedics, Amphia Hospital, Breda, The Netherlands; Pulvertaft Hand Centre, Royal Derby Hospital, Derby, UK

**Keywords:** DASH, Grip strength, Grip strength ratio, Handgrip dynamometry

## Abstract

**Background:**

Grip strength correlates with personal factors such as gender, age and nutritional status and has a good inter-rater reliability. It reflects fairly well how much people can use their hands.

The Disabilities of the Arm, Shoulder and Hand (DASH) Outcome Measure 3 is a 30-item, self-report, questionnaire that reflects the patients’ opinion on their disability due to upper-limb disorders. We assessed if grip strength and grip strength ratio correlate with DASH score.

**Methods:**

In 3 groups (20 healthy volunteers, 17 patients after distal radius fractures, 12 patients with different hand/wrist conditions) grip strength and DASH scores (items 1–21, 22–30 and total) were assessed. To exclude personal factors grip strengths in the injured or non-dominant hand and grip strength ratios (grip strength in the injured or non-dominant hand divided by grip strength in the non-injured or dominant hand) were assessed too. Results were analyzed groups using Pearson Correlation Coefficients and with a multivariate ANOVA.

**Results:**

Grip strength ratio was 0.97 in healthy volunteers, 0.52 in patients after distal radius fracture and 0.74 in patients with various other hand/wrist disorders.

Significant correlations were found between the grip strength ratio and DASH as well as DASH subsections in all groups and between DASH scores and grip strength in some. The correlations between the ratio of the grip strength (GSR) and DASH were much stronger than the correlation between grip strength and DASH. This emphasizes the value of the GSR*.* Age showed no correlation with grip strength ratio using a multivariate ANOVA.

**Conclusion:**

Grip strength ratio correlates well with the DASH score in different hand and wrist conditions. It is a valuable tool to assess patients that speak a different language and have problems with the non-dominant hand and probably easier to follow over time than the DASH score, which is time consuming to fill in and process.

**Electronic supplementary material:**

The online version of this article (doi:10.1186/1471-2474-15-336) contains supplementary material, which is available to authorized users.

## Background

In patients with disorders of the hand and wrist it is important to examine how much the function of the hand and wrist is affected by the condition. Function can be assessed objectively by physical examinations such as range of motion and grip strength and subjectively by questionnaires.

Grip strength in the dominant hand correlates well with certain anthropometric variables such as height, weight, BMI and total arm length and correlates indirectly with certain questionnaires [1–5]. However, it is unknown if it correlates directly with the Disabilities of the Arm, Shoulder and Hand (DASH) Outcome Measure in healthy and injured subjects with various hand and wrist conditions.

The DASH score consists of a 30-item, patient-reported questionnaire designed to measure physical function and symptoms in patients with musculoskeletal disorders of the upper limb ([6], http://www.dash.iwh.on.ca). This score can aid to estimate the disability experienced by patients with hand and wrist disorders and also to monitor changes in symptoms and function over time. It has been shown that this score performs well in both these roles and can be used for research purposes and in clinical practice ([7, 8], Beaton et al., [9]). However, it has been recognised that injuries in the non-dominant hand will not always be picked up fully with DASH, as opposed to the grip strength, which is affected independent on which hand is injured.

To exclude personal factors influencing grip strength, grip strength in the injured or non-dominant hand can be processed into a *grip strength ratio* (grip strength in the injured or non-dominant hand divided by grip strength in the non-injured or dominant hand). This grip strength ratio may be used to evaluate function and symptoms in patients with wrist disorders and this would be a very quick assessment.

The aim of this study is to assess the correlation between grip strength and DASH score in healthy volunteers and patients with hand and wrist disorders. We hypothesize that there is a correlation between grip strength ratio and DASH score.

## Methods

This study involves analysis of grip strength of 37 patients with hand and wrist conditions, who were seen in the outpatient clinic of the department of orthopaedics of the General Hospital of Helsingborg, Sweden, as well as the data collected in 20 healthy volunteers (staff at this hospital). Three groups have been created: Group A consists of 20 healthy volunteers (mean age 42 years, SD 10.2). Group B consists of 17 patients with distal radius fractures undergoing rehabilitation shortly (but not immediately) after plaster removal (mean age 42 years, SD 15.5). Group C consists of 12 patients with different hand and wrist conditions such as osteoarthritis, post-traumatic disorders and tendovaginitis (mean age 52 years, SD 14.5).

Grip strength and DASH score (items 1–21, 22–30 and total) were assessed for correlation with DASH and subgroups. Grip strength was determined three times bilaterally with the Jamar dynamometer (in the second handle position) while the examined person was positioned with the shoulder adducted in zero degrees of rotation, the elbow flexed 90 degrees and the wrist in neutral position [10]. The average of the 3 measurements was used for analysis.

In patients, the grip strength ratio was calculated by dividing the grip strength in the injured hand by the grip strength in the non-injured hand. In the healthy volunteers, this ratio was calculated by dividing the grip strength in the non-dominant hand by the grip strength in the dominant hand.

Results were analyzed using Pearson Correlation Coefficients and a multivariate ANOVA with significance set at p ≤0.05.

This research has adhered to the STROBE guidelines for observational studies. The study was conducted in accordance with the guidelines published by the Swedish Research Council and the International Committee of Medical Journal Editors and supported by the institutional review board of the General Hospital of Helsingborg with written informed consent obtained for publication of this report.

## Results

Grip strength ratio was 0.97 in healthy volunteers, 0.52 in patients after distal radius fracture and 0.74 in patients with various other hand and wrist disorders (Table 1).

**Table 1:** **DASH score, grip strengths and ratio in the groups (mean (SD))**

	Group A healthy volunteers (n =20)	Group B distal radius fractures (n =17)	Group C various hand conditions (n =12)	Entire group (n =49)
Grip strength (kg) dominant or uninjured	44 (12)	41 (11)	44 (15)	43 (12)
Grip strength (kg) non-dominant or injured	43 (12)	21 (10)	30 (15)	32 (16)
Grip strength ratio	0.97 (0.04)	0.52 (0.03)	0.67 (0.23)	0.74 (0.28)
DASH score	1 (2)	37 (21)	26 (17)	20 (22)

Grip strength in the dominant or uninjured hand did not correlate with DASH. Grip strength in the injured hand did correlate with DASH in patients after a distal radius fracture (p <0.05) but not with DASH score in the healthy volunteers and the patients with various hand/wrist conditions. Grip strength ratio correlated significantly with DASH score in all groups (Table 2).

**Table 2:** **Correlations between DASH and grip strength and DASH and grip strength ratio in the groups**

	Group A healthy volunteers (n =20)	Group B distal radius fractures (n =17)	Group C various hand conditions (n =12)	Entire group (n =49)
Grip strength (kg) dominant or uninjured	-.385	.184	.178	.009
Grip strength (kg) non-dominant or injured	-.430	-.502*	-.294	-.633**
Grip strength ratio (%)	-.519*	-.763**	-.784**	-.888**

*correlation between DASH and grip strength (ratio) with level at 0.05.

**correlation between DASH and grip strength (ratio) with level at 0.01.

**Figure 1:**
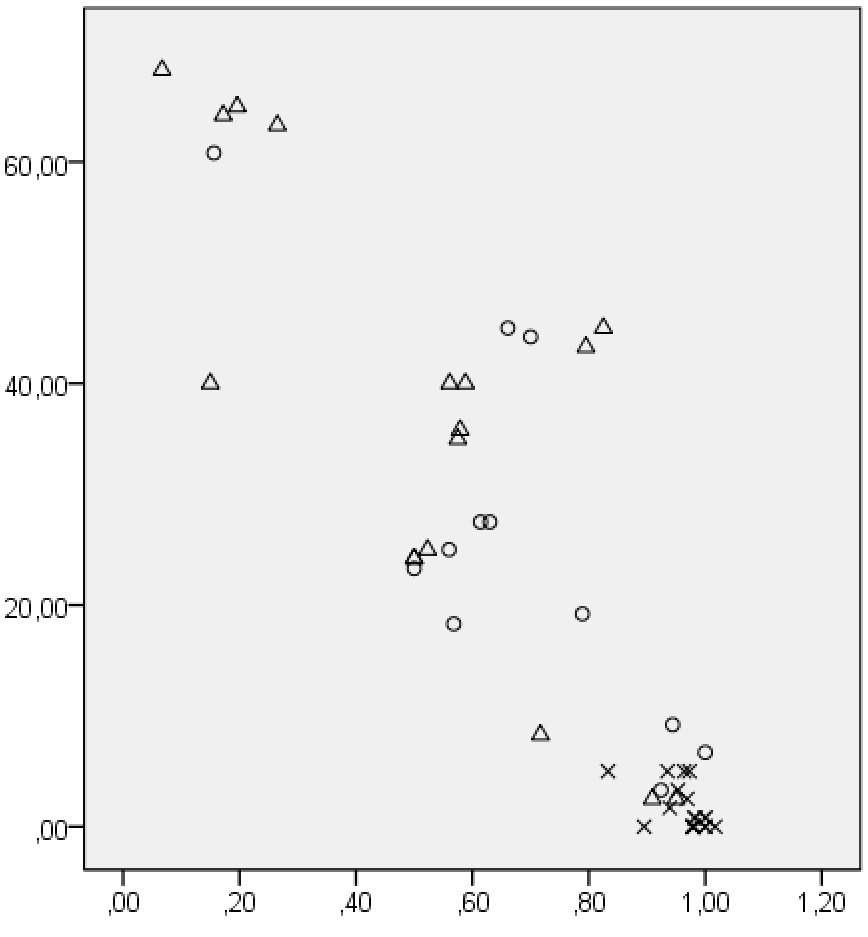
**DASH-score vs Grip strength ratio.** Legends. X-axis: Grip Strength Ratio. Y-axis: DASH score. X healthy controls. Δ patients recovering from radius fracture. O other patients.

The DASH-score in relation to grip strength ratio is displayed in Figure 1. Grip strength ratio correlated significantly with DASH subgroups (Table 3). Grip strength in the injured or non-dominant hand correlated significantly with DASH 1–21 (-.609; p <0.01) and DASH 22–30 (-.610; p <0.01), as well as DASH 1–21 (-.462; p < 0.005) in healthy volunteers and with DASH 22–30 (-.528; p <0.05) in patients after distal radius fracture. No other significant correlations were found.

**Table 3:** **Correlations between DASH subgroups (1–21, 22–30) and grip strength ratio in the groups**

	Group A healthy volunteers (n =20)	Group B distal radius fractures (n =17)	Group C various hand conditions (n =12)	Entire group (n =49)
DASH subgroup 1-21	-.496*	.710**	-.664*	-.844**
DASH subgroup 22-30	-.499*	-.828**	-.674*	-.876**

*correlation between DASH subgroups and grip strength ratio with level at 0.05.

**correlation between DASH subgroups and grip strength ratio with level at 0.01.

With Shapiro-Wilk testing it was shown that the entire group of patients (i.e. the total group without the healthy volunteers) had a normal distribution in grip strength ratio and DASH scores. Subsequently, a multivariate ANOVA showed that age has no influence on grip strength ratio in patients (p = 0.85).

## Discussion

This study shows that the grip strength ratio correlates well with the DASH score in different hand and wrist conditions. Grip strength (ratio) is a valuable tool in all patients but particularly useful to assess patients with intellectual or language difficulties. Furthermore grip strength (ratio) is much faster to process than DASH.

Grip strength ratio is a new ‘tool’. It is the relation of grip strength of the affected hand compared to the unaffected hand or the strength of the non-dominant divided by the dominant hand.

The purpose of the grip strength test is to measure the maximum isometric strength of the hand and forearm muscles. A series of squeezes on the dynamometer in the second handle position is sufficiently accurate to assess grip strength for all subjects. [11]. Grip strength correlates with personal factors such as hand dominance, gender, age and nutritional status (height, weight, BMI) as well as total length [1]. It has a good inter-rater reliability [6] and we think it reflects fairly well how much people can use their hands.

Men have higher values of grip strength compared to women. Of specific importance is the knowledge that right hand and dominant handgrip scores are higher (statistically significant) compared to left hand and non-dominant handgrip scores. The general assumption is that hand dominance vary from 0–10% difference in grip strength with reports that show around 10 percent greater grip strength in the right-handed dominant person and approximately equivalent strength in both hands in left-handed individuals [12–14].

A positive association is also known to exist between height and dominant handgrip strength, while a negative association was observed between age and dominant handgrip strength. A positive association for weight and dominant hand grip strength was statistically significant in men only, whereas a positive, but not significant, association between BMI and dominant hand grip strength was seen in women [2, 15]. Statistically significant lower values in grip strength were seen in the age groups between 65 and 70 years old and 70 to 79 years old when compared with young adult mean values. [4]

It is also known that grip strength is an independent predictor of bone mass in both sexes in children. This relationship between muscle strength and bone mass is systemic. Significant correlations were shown between grip strength and bone mass at hip, spine and whole body in boys (11/12 years old) and girls (10/11 years old) [3].

When grip strength in the injured or non-dominant hand is divided by grip strength in the non-injured or dominant hand a grip strength ratio is determined. This grip strength ratio is independent of the previously mentioned personal factors, and therefore more suitable to assess outcome in less homogenous groups especially in busy clinical settings rather than in a scientific university setting.

Patient questionnaires allow the assessment of outcome without the need for the patient to attend the outpatient clinic.

The Disabilities of the Arm, Shoulder and Hand (DASH) Outcome Measure (ref) is a 30-item, self-report, questionnaire that reflects the patients’ opinion on their disability due to with upper-limb disorders (http://www.dash.iwh.on.ca, [16, 17]).

With the objective to shorten the DASH score to make it more user friendly in clinical practice, the Quick-DASH was developed. This tool consists of 11 instead of 30 items and was also validated for evaluating function and symptoms in patients with wrist disorders [18]. Independent on DASH or Quick-DASH there is a problem when assessing conditions affecting the non-dominant hand, which is an important downside with DASH.

It would be easier however if patients could be evaluated using only grip strength. In certain groups of patients DASH was found to correlate with grip strength or other clinical and radiological findings [19, 20]. In a study correlating DASH to the Levine-Katz questionnaire using clinical findings and pinch and grip strength in patients with ulnar neuropathology, it was found that a high correlation between DASH scores, symptom severity and functional status of patients with ulna neuropathology existed [19]. Another indirect correlation between DASH and grip strength was found in patients after distal radius fractures. Certain radiological parameters of malunion of the distal radius (radial shortening of more than 2 mm, dorsal angulation of more than 15° and radial angulations of more than 10° each) are significantly associated with poorer DASH scores in patients with healed unilateral distal radius fractures. In these patients reduced grip strength, extension and ulnar deviation correlated with a poorer DASH score, whereas the radiological and objective physical results (grip strength and range of motion) were associated with a better DASH score [20].

In multivariate analysis some patient questionnaires (SODA, AIMS) were found to correlate with grip strength and certain clinical features ([5].

In certain groups DASH was also found to correlate with grip strength or other clinical and radiological findings [19, 20]. To our knowledge, however, there are no reports that have directly correlated the DASH score with grip strength as well as the grip strength ratio between the two wrists, in healthy volunteers and groups of patients with different wrist conditions.

Patient questionnaires allow the assessment of outcome without the need for the patient to attend the outpatient clinic. This saves time, but the forms still need to be sent out, processed, monitored and evaluated. Although in general a patient questionnaire is a very efficient and unbiased way of collecting information on outcome for routine use, grip strength may be an easier tool in patients with different hand and wrist conditions. Since grip strength is related to personal factors such as age, gender, hand dominance and weight, we sought for a different modality of grip strength to correlate with the outcome of a patient questionnaire, because these questionnaires cannot be used in patients with intellectual or language difficulties, a growing group in our cosmopolitan world. It is not our intention to replace DASH with grip strength ratio but to use grip strength ratio when the use of a PROM is not possible or suitable.

We chose a group with healthy volunteers, a homogenous group of patients with injuries (patients shortly after treatment of a distal radius fracture with a plaster) and a non-homogeneous group with various hand and wrist conditions to gain as much information as possible about the correlation between grip strength, grip strength ratio and DASH score.

We assumed a close relationship between full grip strength and a healthy DASH score, which we have shown in the healthy volunteers. In addition we wanted to see whether the important group of patients with distal radius fractures demonstrated a similar relationship, which we have shown. We finally looked into a group of various hand and wrist conditions and demonstrated that the ratio correlates with DASH in this group as well.

In patients with distal radius fractures and healthy volunteers a significant correlation was found between grip strength and DASH score for certain subgroups. However, the correlations between the ratio of the grip strength (GSR) and DASH were much stronger and significant in all cases. This emphasizes the value of the GSR. Furthermore, as grip strength ratio is shown to correlate well with the outcome of the DASH score in all 3 groups (patients and healthy volunteers), including the group with various hand and wrist conditions, one might expect this correlation to be present in more groups with more homogeneous conditions, and therefore be useful for clinical practice in patients with only one extremity involved. As mentioned previously a small difference in grip strength ratio might be found between right-handed and left-handed dominant individuals.

Since this is only a small study we advise that the correlation between grip strength ratio and hand dominance as well as other hand/wrist conditions will be subject of further studies.

## Conclusion

Grip strength ratio correlates well with the DASH score in different hand and wrist conditions. It is a valuable tool to assess patients that speak a different language and probably easier to follow over time than the DASH score especially in a busy clinical setting. We do not feel it can replace DASH in research projects at this point.

## Authors’ original submitted files for images

Below are the links to the authors’ original submitted files for images. Authors’ original file for figure 1

## Abbreviations

AIMS: Arthritis impact measurement scales
BMI: Body mass index
DASH: Disabilities of the arm, shoulder and hand
GSR: Grip strength ratio
SD: Standard deviation
SODA: Sequential occupational dexterity assessment.
