# Supplementary figures and images for: Grip strength ratio: a grip strength measurement that correlates well with DASH score in different hand/wrist conditions
Source: BMC Musculoskelet Disord. 2014 Oct 6;15:336. doi: 10.1186/1471-2474-15-336 (PMC4197251; doi:10.1186/1471-2474-15-336)

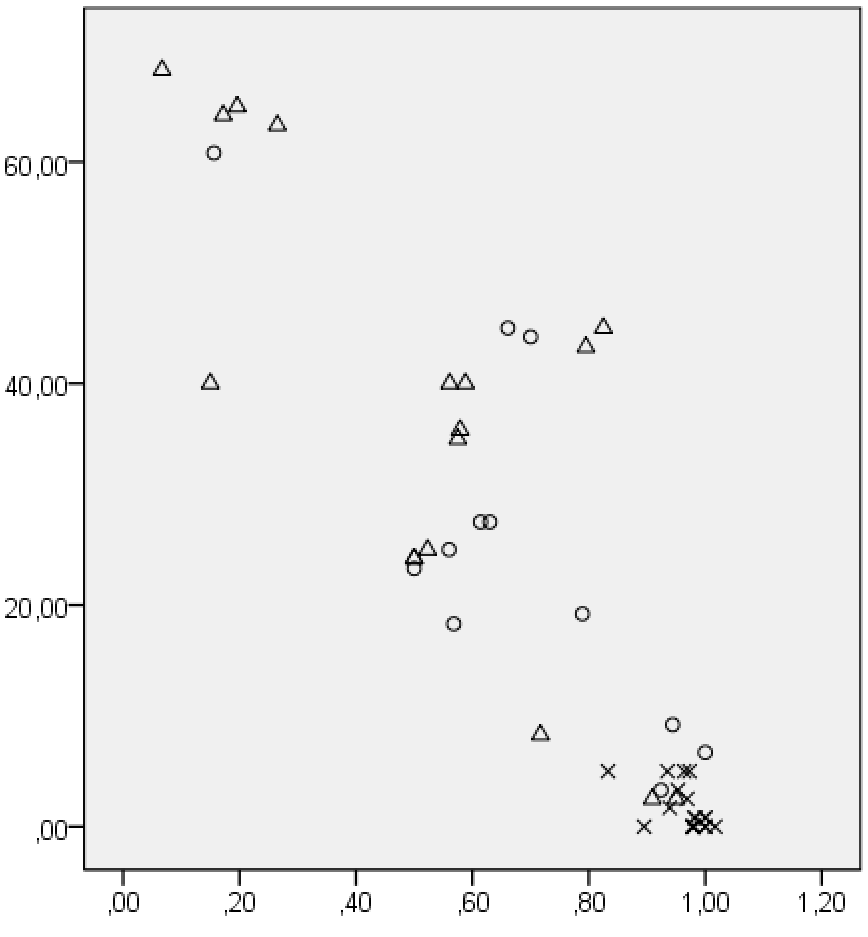

Supplement: Supplementary file 1 — Authors’ original file for figure 1 [file 12891_2014_2273_MOESM1_ESM.tif]
